# Common variants in *LEPR*, *IL6*, *AMD1,* and *NAMPT* do not associate with risk of juvenile and childhood obesity in Danes: a case–control study

**DOI:** 10.1186/s12881-015-0253-3

**Published:** 2015-11-11

**Authors:** Mette Hollensted, Tarunveer S Ahluwalia, Christian Theil Have, Niels Grarup, Cilius Esmann Fonvig, Tenna Ruest Haarmark Nielsen, Cæcilie Trier, Lavinia Paternoster, Oluf Pedersen, Jens-Christian Holm, Thorkild I A Sørensen, Torben Hansen

**Affiliations:** The Novo Nordisk Foundation Center for Basic Metabolic Research, Section of Metabolic Genetics, Faculty of Health and Medical Sciences, University of Copenhagen, DIKU Building, Universitetsparken 1, 2100 Copenhagen, Denmark; COPSAC, Copenhagen Prospective Studies on Asthma in Childhood, Herlev and Gentofte Hospital, University of Copenhagen, Ledreborg Allé 34, DK-2820 Copenhagen, Denmark; Steno Diabetes Center, Gentofte, Denmark; The Children’s Obesity Clinic, Department of Pediatrics, Copenhagen University Hospital Holbæk, Holbæk, Denmark; MRC Integrative Epidemiology Unit, School of Social and Community Medicine, University of Bristol, Bristol, UK; Institute of Preventive Medicine, Bispebjerg and Frederiksberg Hospital, The Capital Region, Copenhagen, Denmark

**Keywords:** Childhood obesity, Juvenile obesity, Single nucleotide polymorphism, Case-control study, Body mass index, BMI, Obesity

## Abstract

**Background:**

Childhood obesity is a highly heritable disorder, for which the underlying genetic architecture is largely unknown. Four common variants involved in inflammatory-adipokine triggering (*IL6* rs2069845, *LEPR* rs1137100, *NAMPT* rs3801266, and *AMD1* rs2796749) have recently been associated with obesity and related traits in Indian children. The current study aimed to examine the effect of these variants on risk of childhood/juvenile onset obesity and on obesity-related quantitative traits in two Danish cohorts.

**Methods:**

Genotype information was obtained for 1461 young Caucasian men from the Genetics of Overweight Young Adults (GOYA) study (overweight/obese: 739 and normal weight: 722) and the Danish Childhood Obesity Biobank (TDCOB; overweight/obese: 1022 and normal weight: 650).

Overweight/obesity was defined as having a body mass index (BMI) ≥25 kg/m^2^; among children and youths, this cut-off was defined using age and sex-specific cut-offs corresponding to an adult body mass index ≥25 kg/m^2^. Risk of obesity was assessed using a logistic regression model whereas obesity-related quantitative measures were analyzed using a general linear model (based on *z-*scores) stratifying on the case status and adjusting for age and gender. Meta-analyses were performed using the fixed effects model.

**Results:**

No statistically significant association with childhood/juvenile obesity was found for any of the four gene variants among the individual or combined analyses (rs2069845 OR: 0.94 CI: 0.85–1.04; rs1137100 OR: 1.01 CI: 0.90–1.14; rs3801266: 0.96 CI: 0.84–1.10; rs2796749 OR: 1.02 CI: 0.90–1.15; *p* > 0.05). However, among normal weight children and juvenile men, the *LEPR* rs1137100 A-allele significantly associated with lower BMI (β = −0.12, *p* = 0.0026).

**Conclusions:**

The *IL6*, *LEPR*, *NAMPT*, and *AMD1* gene variants previously found to associate among Indian children did not associate with risk of obesity or obesity-related quantitative measures among Caucasian children and juvenile men from Denmark.

## Background

The burden of childhood obesity has increased dramatically within the past two decades and as of today one in every three children (6–9 years old) within the European Union is overweight or obese [1]. Childhood obesity is associated with numerous related complications and serious diseases later in life, including cardiovascular disease [2], type 2 diabetes, several cancers and rheumatologic disorders, as well as social stigmatization and psychological health problems [3, 4]. Although the recent increase in the prevalence of childhood obesity is most likely a result of environmental changes, a genetic component contributing to the risk of obesity is also evident from twin studies [5, 6]. To date, genome-wide association studies (GWAS) have identified well over 100 adiposity susceptibility loci [7, 8], however, only a limited number of these have been implicated in childhood obesity [9–11].

Recent studies suggest that white adipose tissue inflammation has an emerging role in obesity pathophysiology [12]. White adipose tissue plays an important role in the production of bioactive adipokines that possess immune-regulatory properties in addition to regulation of systemic metabolism. A recent study explored the role of genes encoding adipokines and inflammatory markers (*interleukin 6, IL6*; *leptin receptor*, *LEPR*, and *nicotinamide phosphoribosyltransferase, NAMPT*) in 2,261 normal weight and 907 overweight/obese children of Indian origin (age range: 11–17 years). Overweight/obesity was defined using age and sex-specific cut-offs corresponding to a BMI ≥25 kg/m^2^ in adults [13]. When employing a two stage study design, all three variants (*IL6*, *LEPR*, and *NAMPT*) were found to associate with an increased prevalence of childhood obesity (OR_combined_ >1.35) and related quantitative measures (BMI, weight, waist and hip circumference, *p ≤* 2.0 × 10^−3^) [14].

A follow-up study in the Indian cohort reported the *AMD1*gene *(adenosylmethionine decarboxylase 1)* single nucleotide polymorphism (SNP) rs2796749 G allele to be associated with childhood obesity (OR = 1.35; *p* = 1.9 × 10^−6^) and related quantitative measures (BMI, weight, height, waist and hip circumference, as well as plasma leptin levels (*p* ≤ 6.7 × 10^−4^) [15]. *AMD1* encodes an important intermediate enzyme involved in the homocysteine pathway.

Previous studies examining the effect of *IL6, LEPR, NAMPT*, and *AMD1* variants on risk of childhood obesity in Caucasians are very limited and have generally been small and statistically underpowered [16, 17]. Thus, the aim of the present study was to replicate the findings by Tabassum et al. [14, 15] by examining the effect of four SNPs: *IL6* rs2069845, *LEPR* rs1137100, *NAMPT* rs3801266, and *AMD1* rs2796749 on the risk of childhood/juvenile obesity and anthropometric traits (BMI, height, and weight) in the two Danish study populations comprising cases of childhood or juvenile obesity and normal weight controls.

## Methods

The present study was conducted in accordance with the Helsinki declaration and approved by the Danish Data Protection Agency and by the regional scientific ethics committees. Prior to this study, all participants aged 18 or parents of younger children signed an informed written consent.

### Study participants

#### GOYA male

In the recruitment of these participants, a case-cohort sampling design was employed in which cases were defined as the individuals with the upper-most extreme BMI in the cohort, and controls were randomly selected from the same cohort [18].

During 1943–1977, 362200 Caucasian men with a mean age of ~20 years were examined at draft boards in the area of Copenhagen, Denmark. At the draft board examinations, standing height (without shoes) and weight (in underwear only) were measured. Obesity was defined as 35 % overweight relative to a local standard in use at the time (mid 1970’s). This definition corresponds to a BMI ≥31.0 kg/m^2^, which proved to be above the 99th percentile. All obese men (*n* = 1930) and a randomly selected control group (*n* = 3601), comprising one percent of all participants, were sampled on the basis of the records from the draft board examinations.

In 1991–1994, at the mean age of 46 years, all obese men and 50 % of the randomly selected young men from the study population were invited for a follow-up survey (753 obese and 879 control men attended), at which time non-fasting venous blood samples were obtained [19]. By using a sampling fraction of 0.5 % (50 % of 1 %), the included controls effectively represent approximately 158000 men among whom the case group was the most obese. Criteria for invitation to follow-up surveys and participation have been described elsewhere [20].

In the current study, while replicating the original studies by Tabassum et al. [14, 15], we adopted the BMI cut-off corresponding to overweight/obesity defined as BMI ≥25 kg/m^2^, and 1461 (*n*_overweight/obese_: 739 and *n*_normal weight_: 722) men had complete genetic and phenotypic information representing the GOYA (male) cohort.

#### The Danish Childhood Obesity Biobank (TDCOB)

All children were recruited through The Children’s Obesity Clinic, Copenhagen University Hospital Holbæk, Denmark. Obese children, defined by a BMI above the 90^th^ percentile for age and sex according to Danish standard BMI charts [21], aged 3 to 18 years were treated using the Children’s Obesity Clinic’s Treatment Protocol [22]. In addition, an age and gender matched control group was recruited from schools and high schools in the surrounding area.

Height and weight were measured to the nearest 0.1 cm and 0.1 kg, respectively, using an integrated calibrated weight and stadiometer (ADE, Modell MZ10023, Germany) while wearing light indoor clothing. Danish standard BMI charts [21] were used to calculate BMI (kg/m^2^) into BMI z-scores. Prior to analysis, children with syndromes (e.g. Turner syndrome, Down’s syndrome) or other clinical diagnoses known to associate with obesity were excluded.

A total of 1672 children (*n*_overweight/obese_: 1022 and *n*_normal weight_: 650), from the TDCOB cohort participated in the current study.

Clinical characteristics for both study populations are listed in Table 1.

**Table 1:** Clinical characteristics of participants from the GOYA (male) and the TDCOB study

Characteristics	GOYA	TDCOB
*N*_total_ (%men)	1461 (100)	1672 (41.9)
*N*_overweight/obese_/*N*_normal weight_	739/722	1022/650
Age_overweight/obese_	19.9 ± 1.9	11.7 ± 3.2
Age_normal weight_	19.9 ± 1.7	12.7 ± 3.3
BMI_overweight/obese_	32.6 ± 3.0	27.2 ± 5.8
*z*BMI_overweight/obese_	6.4 ± 1.6	2.8 ± 0.70
BMI_normal weight_	21.1 ± 1.7	18.4 ± 2.7
*z*BMI_normal weight_	0.04 ± 1.0	0.11 ± 0.89
Height_overweight/obese_	177.3 ± 6.1	153.3 ± 16.3
*z*Height_overweight/obese_	0.10 ± 0.9	0.96 ± 0.98
Height_normal weight_	176.7 ± 6.6	155.4 ± 16.3
*z*Height_normal weight_	0.007 ± 1.0	0.66 ± 0.99
Weight_overweight/obese_	102.7 ± 11.5	66.5 ± 25.6
*z*Weight _overweight/obese_	5.28 ± 1.6	4.0 ± 0.52
Weight_normal weight_	66.1 ± 6.9	45.9 ± 14.5
*z*Weight_normal weight_	0.011 ± 1.0	3.86 ± 0.51

Data are means ± standard deviations. Individuals were classified as being overweight/obese or normal weight using age and sex-specific cut-offs corresponding to a BMI ≥25 kg/m^2^ in adults

### Genotyping

#### GOYA (Male)

Genotyping on the Illumina Human 610 k quad bead chip was performed at the Centre National de Génotypage, Evry, France. Genotypes were called using the Gen Call algorithm from Genome Studio package. In brief, the data quality control included 1) exclusion of SNPs with a minor allele frequency <1% and >5 % missing genotypes, 2) exclusion of those failing Hardy Weinberg equilibrium (HWE, *p* < 10^−7^), and 3) including only European individuals (clustering with the HapMap CEU population (Utah residents with Northern and western European ancestry)). Further checks on heterozygosity, sample duplicates, individual relatedness, or with sex discrepancy were made. Imputation was carried out to HapMap release 22 (CEU individuals) using Mach 1.0, Markov Chain Haplotyping [23].

Finally, we extracted genotypes for two SNPs (*LEPR* rs1137100 and *AMD1* rs2796749) available on the SNP array, whereas the other two (*IL6* rs2069845, *NAMPT* rs3801266) were imputed with an r^2^ ≥ 0.8.

#### TDCOB

The SNPs were genotyped on Illumina Infinium HumanCoreExome Beadchip platform at the Novo Nordisk Foundation Centre for Basic Metabolic Research’s laboratory at Symbion, Copenhagen, Denmark. Genotypes were called with Illumina standard pipeline in Genome Studio. The data quality control involved 1) HWE filter: variants with *p*_HWE_ >0.005 were retained, 2) ethnicity checks using principal component analyses were performed, and individuals identified as ethnic outliers or with extreme positive or negative inbreeding coefficients were removed. Checks on heterozygosity, sample duplicates, or with sex discrepancy were made. Additional genotypes were imputed into 1000 genomes phase 1 [24] using impute2 [25]. All variants examined in the current study were imputed with high quality (info >0.95). In case of siblings, only the sibling with the best overall genotype call-rate was retained in the study (regardless of gender).

### Statistical analyses

The quantitative measures were transformed to z-scores (standard deviations (sd) from the study reference group (controls) mean) using the equation z (z-score) = (x − μ)/σ where x is the raw measure, μ is the mean value for the study reference group, and σ is the sd for that reference group. Logistic regression was used to compare allele frequencies in the dichotomized case–control groups whereas a general linear model was applied to test quantitative traits using an additive genetic model while adjusting for age and gender. For these analyses, we adopted both the age and gender dependent BMI cut-off corresponding to an adult BMI ≥25 kg/m^2^ provided by Cole et al. [13] as per the studies by Tabassum et al. [14, 15], as well as a BMI cut-off of 31 kg/m^2^, taking into account the original GOYA study design. No transformation of traits was performed as they followed normal distributions within case or control groups. Linear regression analyses were performed separately in the case and cohort groups owing to the trait distribution as per study design. A *p*-value of less than 0.05 was considered statistically significant. Association tests were conducted on the imputed genotype likelihoods using the frequentist score method of the SNPtest tool (version 2.5) with a standard additive model among TDCOB children and using R statistical software (version 2.12.1) [26] in the GOYA males.

We combined the individual study results (obesity risk and quantitative trait (*z*BMI, *z*Weight, *z*Height) association results) to perform a fixed effects (inverse variance) meta-analysis where weights are proportional to the squared standard error of the effect estimates, as implemented in the METAL software [27]. The heterogeneity in effect sizes across the two participating cohorts was evaluated using the chi-square test for heterogeneity (*I*^2^) also through METAL.

### External data look-up

External data from published GWAS meta-analyses on BMI was contributed by GIANT consortia investigators and was freely accessible [28].

## Results

### Association with obesity

We found no association between the four genetic loci (*IL6* rs2069845, *LEPR* rs1137100, *NAMPT* rs3801266, and *AMD1* rs2796749) and prevalence of obesity (corresponding to an adult BMI cut-off of 25 kg/m^2^) among Danish young men or children (Table 2). We also checked for associations taking into account the original GOYA study design (corresponding to a BMI cut-off of 31 kg/m^2^) but the results remained insignificant (*p* > 0.05; data not shown).

**Table 2:** Association with obesity: Case–control analysis in GOYA (male) and TDCOB study participants

SNP	Locus	Alleles (effect/other)	GOYA (*n* = 1461)				TDCOB (*n* = 1672)				Combined (*n* = 3133)			
			*n* (cases/controls)	*EAF* (cases/controls)	*p*	OR (95 % CI)	*n* (cases/controls)	*EAF* (cases/controls)	*p*	OR (95 % CI)	*n* (cases/controls)	*p*	OR (95 % CI)	I^2^ (*p*_*HET*_)
rs2069845	*IL6*	G/A	739/722	0.49/0.49	0.88	0.98 (0.85–1.14)	1022/650	0.47/0.49	0.13	0.88 (0.76–1.01)	1761/1372	0.24	0.94 (0.85–1.04)	0 (0.35)
rs1137100	*LEPR*	A/G	739/722	0.74/0.74	0.69	0.96 (0.81–1.14)	1022/650	0.75/0.74	0.42	1.05 (0.90–1.23)	1761/1372	0.46	1.01 (0.90–1.14)	0 (0.40)
rs3801266	*NAMPT*	T/C	739/722	0.82/0.83	0.96	0.99 (0.82–1.20)	1022/650	0.83/0.84	0.47	0.92 (0.76–1.11)	1761/1372	0.58	0.96 (0.84–1.10)	0 (0.63)
rs2796749	*AMD1*	G/C	739/722	0.78/0.78	0.94	1.0 (0.84–1.19)	1022/650	0.79/0.78	0.59	1.03 (0.87–1.22)	1761/1372	0.68	1.02 (0.90–1.15)	7 (0.73)

Cases and controls were defined using age and sex-specific cut-offs corresponding to a BMI ≥25 kg/m^2^ in adults. *p*-values are for an additive genetic model and are adjusted for age

*EAF* Effect allele frequency, *BMI* body mass index, *CI* confidence interval, *HET* heterogeneity, *OR* Odds Ratio, *SNP* single nucleotide polymorphism

Furthermore, no statistically significant association with obesity was found for either of the variants in the combined analyses (rs2069845: OR [95 % CI] = 0.94 [0.85–1.04], *p*_combined_ = 0.24; rs1137100: OR [95 % CI] = 1.01 [0.90–1.14], *p*_combined_ = 0.46; rs3801266: OR [95 % CI] = 0.96 [0.84–1.10], *p*_combined_ = 0.58; and rs2796749: OR [95 % CI] = 1.02 [0.90–1.15], *p*_combined_ = 0.68; Table 2).

### Association with obesity-related quantitative traits

We then examined the four SNPs for association with obesity-related traits (zBMI, zHeight, and zWeight) in the normal weight and overweight/obese groups separately.

Among the normal weight juvenile men, the *LEPR* rs1137100 A-allele significantly associated with zBMI (β = −0.17, *p*_GOYA_ = 0.0036), and this association remained significant in the combined analyses (β = −0.12, *p*_combined_ = 0.0026; Table 3).

**Table 3:** Association with quantitative traits in 1372 normal weight individuals

SNP	Locus	Alleles (effect/other)	Effect allele frequency	GOYA (*n* = 722)			TDCOB (*n* = 650)			Combined (*n* = 1372)			
				β	SE	*p*	β	SE	*p*	β	SE	*p*	*I* ^2^ (*p*_*HET*_)
*z*BMI													
rs2069845	*IL6*	G/A	0.49	0.04	0.05	0.34	−0.02	0.05	0.62	0.01	0.04	0.76	3 (0.31)
rs1137100	*LEPR*	A/G	0.74	−0.17	0.06	**0.0036**	−0.08	0.06	0.15	−0.12	0.04	**0.0026**	20 (0.26)
rs3801266	*NAMPT*	T/C	0.83	−0.03	0.07	0.58	0.04	0.07	0.51	0.01	0.05	0.93	0 (0.39)
rs2796749	*AMD1*	G/C	0.78	0.03	0.06	0.60	−0.16	0.06	**0.0077**	−0.06	0.04	0.12	80 (0.03)
*z*Height													
rs2069845	*IL6*	G/A	0.49	0.02	0.05	0.65	−0.07	0.06	0.19	−0.02	0.04	0.56	36 (0.20)
rs1137100	*LEPR*	A/G	0.74	0.01	0.06	0.63	−0.08	0.06	0.17	−0.04	0.04	0.36	5 (0.30)
rs3801266	*NAMPT*	T/C	0.83	0.07	0.07	0.32	−0.12	0.08	0.12	−0.02	0.05	0.75	69 (0.07)
rs2796749	*AMD1*	G/C	0.78	−0.12	0.06	**0.046**	0.10	0.07	0.15	−0.02	0.05	0.64	82 (0.01)
*z*Weight													
rs2069845	*IL6*	G/A	0.49	0.06	0.05	0.29	−0.05	0.03	**0.047**	−0.03	0.02	0.19	69 (0.06)
rs1137100	*LEPR*	A/G	0.74	−0.14	0.06	**0.017**	−0.02	0.03	0.44	−0.05	0.03	0.08	68 (0.07)
rs3801266	*NAMPT*	T/C	0.83	0.02	0.07	0.82	−0.01	0.04	0.79	−0.01	0.03	0.89	0 (0.75)
rs2796749	*AMD1*	G/C	0.78	−0.07	0.06	0.29	−0.01	0.03	0.95	−0.01	0.03	0.66	0 (0.33)

Effect sizes (β), standard error (SE) and *p*-values are for an additive genetic model based on *z*-scores of each trait and are adjusted for age. Effect allele frequency is the mean frequency observed for the GOYA and TDCOB cohort control individuals. *P*_*add*_ < 0.05 is significant and is shown in bold. Values of *I* ^2^ are percentages

*BMI* body mass index, *CI* confidence interval, *HET* heterogeneity, *SE* standard error, *SNP* single nucleotide polymorphism

Furthermore, we observed some cohort specific associations that did not reach statistical significance in the combined analyses. Among GOYA, the *LEPR* SNP associated with reduced zWeight (β = −0.12, *p*_GOYA_ = 0.046) whereas the *AMD1* rs2796749 associated with zHeight (β = −0.14, *p*_GOYA_ = 0.017; Table 3). While taking the original study design for GOYA into account (BMI cut-off of 31 kg/m^2^), we found similar associations for *LEPR* rs1137100 with zBMI and zWeight (β_GOYA_zBMI_ = −0.14, *p*_GOYA_zBMI_ = 0.014; and β_GOYA_zWeight_ = −0.11, *p*_GOYA_zWeight_ = 0.045) whereas non-significant for *AMD1* rs2796749 and zHeight (*p* > 0.05) (data not shown).

In TDCOB, *AMD1* rs2796749 was found to associate with zBMI (β = −0.16, *p*_TDCOB_ = 0.0077) while *IL6* rs2069845 modestly associated with zHeight (β = −0.05, *p* = 0.047; Table 3).

Among the overweight/obese individuals, no significant association with zBMI, zWeight or zHeight was identified in either of the examined cohorts or in the combined analysis (Table 4). A *p*-value corresponding to each chi-square test for heterogeneity has also been indicated (*P*_*HET*_) in the results tables (Tables 2, 3, and 4).

**Table 4:** Association with quantitative traits in 1761 overweight/obese individuals

SNP	Locus	Alleles (Effect/other)	Effect allele frequency	GOYA (*n* = 739)			TDCOB (*n* = 1022)			Combined (*n* = 1761)			
				β	SE	*p*	β	SE	*p*	β	SE	*p*	*I* ^2^ (*p*_*HET*_)
*z*BMI													
rs2069845	*IL6*	G/A	0.48	0.02	0.09	0.78	0.01	0.03	0.90	0.01	0.03	0.84	0 (0.83)
rs1137100	*LEPR*	A/G	0.74	0.11	0.10	0.28	−0.01	0.03	0.71	0.01	0.03	0.99	20 (0.26)
rs3801266	*NAMPT*	T/C	0.83	−0.09	0.12	0.45	−0.05	0.04	0.19	−0.06	0.04	0.13	0 (0.77)
rs2796749	*AMD1*	G/C	0.78	−0.11	0.11	0.31	−0.06	0.04	0.08	−0.07	0.04	0.05	0 (0.69)
*z*Height													
rs2069845	*IL6*	G/A	0.48	0.04	0.05	0.39	−0.01	0.04	0.79	0.01	0.03	0.70	0 (0.41)
rs1137100	*LEPR*	A/G	0.74	0.05	0.05	0.35	−0.05	0.05	0.28	−0.01	0.04	0.89	49 (0.16)
rs3801266	*NAMPT*	T/C	0.83	−0.04	0.06	0.54	−0.02	0.06	0.70	−0.03	0.04	0.48	0 (0.85)
rs2796749	*AMD1*	G/C	0.78	0.05	0.06	0.43	−0.04	0.05	0.51	0.01	0.04	0.94	2 (0.31)
*z*Weight													
rs2069845	*IL6*	G/A	0.48	0.07	0.09	0.41	−0.02	0.02	0.46	−0.01	0.02	0.61	0 (0.33)
rs1137100	*LEPR*	A/G	0.74	0.13	0.10	0.19	0.02	0.03	0.45	0.03	0.03	0.28	11 (0.28)
rs3801266	*NAMPT*	T/C	0.83	−0.11	0.11	0.33	0.03	0.03	0.38	0.02	0.03	0.54	24 (0.25)
rs2796749	*AMD1*	G/C	0.78	−0.03	0.11	0.75	0.02	0.03	0.56	0.01	0.03	0.63	0 (0.65)

Effect sizes (β), standard error (SE) and *p*-values are for an additive genetic model based on *z-*scores of each trait and is adjusted for age. Effect allele frequency is the mean frequency observed for the GOYA and TDCOB cohort obese individuals. *P*_*add*_ < 0.05 is significant. Values of *I* ^2^ are percentages

*Add* additive, *BMI* body mass index, *CI* confidence interval, *HET* heterogeneity, *SE* standard error, *SNP* single nucleotide polymorphism

## Discussion

Common variations in four genes involved in inflammatory and homocysteine pathways *(IL6*, *LEPR*, *NAMPT*, and *AMD1)* have recently been shown to associate with childhood obesity among children of Indian origin [14, 15]. We aimed to replicate these findings, however, we found none of the variants to exert a strong effect on risk of obesity among Danish children and juveniles (OR ≤1.15, as based on 95 % CI, Table 2). We did, however, observe a modest association between the *LEPR* variant and lowered BMI among non-obese Danish children and young men, albeit the directionality of these associations was opposite to what was observed among Indian children [14]. In the studies by Tabassum et al., *IL6, LEPR,* and *NAMPT* variants also associated with hip and waist circumference [14, 15], but unfortunately, information on these traits were not available in the present study.

LEPR is involved in the regulation of fat metabolism, and although mutations in *LEPR* robustly associate with monogenic forms of obesity [29, 30], studies focusing on complex forms of obesity report conflicting results for several variants, including Q223R, K109R and K656N, in adults [31–33]. Reported *LEPR* associations with obesity among children and adolescents have also been inconsistent: Mexican (*n* = 128) and Polish (*n* = 142) studies found no association between *LEPR* rs1137100 [K109R] and obesity [16, 34], whereas a small study of 136 Japanese children found the rs1137100 A-allele to associate with increased risk of being overweight [35]. In the current study, the rs1137100 A-allele associated significantly with a lowered BMI but not with obesity. However, this result was primarily driven by the normal weight juvenile men and not by the children. These inconsistent findings could imply that the effects of rs1137100 may get modified by additional genetic or environmental factors which may vary between populations. Recent studies in Europeans have suggested obesity related measures to be influenced by interactions between *LEPR* polymorphisms and a polymorphism of the tyrosine phosphatase 1B [36] and ponderal index [37] and future studies focusing on such “gene-gene” and “gene-environment” interactions are thus warranted.

In the current study, no association with obesity was identified for the *IL6* variant in the individual cohorts or in the combined analysis. Previous studies examining the putative association between variations in the promoter region of *IL6* and risk of obesity in adults have yielded contradictive results [32, 38–40], and studies among children are limited. Reports on Mexican adolescents (age range: 14–19 years) found *IL6* variants (rs1800795, rs1800796, and rs1800797) to associate with quantitative obesity-related traits and prevalence of obesity [41]. Similarly, a study on Greek primary school children (*n* = 184) reported rs1800795 to associate with obesity-related traits [42]. In the HapMap database, rs1800795 and rs1800797 (for CEU population) are both in relatively high linkage disequilibrium (LD) with rs2069845 (r^2^ > 0.83, D’ > 0.93) which was examined in the present study. The inconsistency between study results may be due to differences in age or gender stratification in the examined study populations, and/or it may relate to statistical power issues.

Variations in *NAMPT* have in recent studies in German and Brazilian children and adolescents not associated with risk of obesity [17, 43] and the similar was observed in the current study involving Danes. However, a French study including children and adults reported a rare *NAMPT* variant (rs10487818) associating with decreased risk of obesity [44]. Since the rare variant (rs10487818) examined among French [44] was unavailable in our study and in the Exome variant server [45], it may represent a population-specific signal.

Albeit we found no association between *AMD1* rs2796749 and risk of obesity, the *AMD1* (rs2796749) G-allele strongly correlated with a lower zBMI among the TDCOB non-obese children and borderline with the TDCOB obese children but not among the GOYA men, suggesting some protective effect of *AMD1* SNP on BMI among children but not young men. Again, it is noteworthy that the *AMD1* G-allele associated with a lower zBMI among Danish TDCOB children whereas directionality of effect among Indian children was opposite. As the first study to report an association between an *AMD1* variant and childhood obesity, Tabassum et al. identified a relatively high OR (1.35, *p* = 1.9 × 10^−6^) for *AMD1* rs2796749 [11], perhaps reflecting a case of “winner’s curse”, or it may indicate that the effect of *AMD1* rs2796749 is population-specific.

All of the four SNPs examined in the present study were included in both a large meta-analysis (*n* = 249796) of European-ancestry-based GWAS [46], and a more recent combined GWAS and Metabochip meta-analysis (*n* = 339224) [8]. Both studies were performed in adults, and no statistically significant association with BMI was observed for any of the variants [8, 46], suggesting that none of the four variants significantly influence the risk of obesity in adulthood and that putative effects may be exerted only in childhood. Although the GOYA study participants were sampled at ~20 years of age, the group of overweight/obese individuals already had a much increased BMI at age seven years, compared to the population sample and this deviation increased as the children grew older [47]. However, BMI data from age seven years were only available in a subset (n ~ 172 in each group) compared to available data at mean age 20 years (n ~ 722 in each group). For that reason we define the individuals with data at age 20 years as juvenile onset obese/overweight cases and controls, in the current study. Moreover, as our statistical power to detect the large ORs reported by Tabassum and colleagues was very high (>90 % for variants with risk allele frequencies >0.40, data not shown), our study design should have enabled us to identify a large effect of these variants on childhood/juvenile obesity, if it had been exerted among Danish individuals.

The identified differences between the current study results and those reported among Indian children [14, 15] may be due to the following reasons: firstly, the observed phenotype at a given BMI cutoff is known to differ between various ethnic populations, and risk factors for cardiovascular disease can be identified at a lower BMI in Asian and Indian populations compared to European [48]. This suggests that applying the same BMI cut-off of 25 kg/m^2^ in both, the Indian and Danish populations may confound the association. Furthermore, genotype frequencies and LD patterns may differ between the populations, and environmental factors such as lifestyle may also modify the effects exerted by genetic variants. Finally, population stratification may also play a role. Genetic marker based clustering analyses have therefore been applied to exclude ethnic outliers in the current study as well by Tabassum and colleagues [14, 15] ruling out the possibility that major population stratification plays a role for the observed differences between the two studies.

## Conclusions

The current study illustrates that four variants (*IL6* rs2069845, *LEPR* rs1137100, *NAMPT* rs3801266, and *AMD1* rs2796749) implicated in childhood obesity among Indians are not strongly associated with obesity among Danish children and juveniles.

## Abbreviations

Add: additive
AMD1: adenosylmethionine decarboxylase 1
BMI: body mass index
CEU: Utah residents with Northern and western European ancestry
CI: confidence interval
GOYA: Genetics of Overweight Young Adults
GWAS: genome-wide association study
HET: heterogeneity
HWE: Hardy Weinberg equilibrium
IL6: interleukin 6
LD: linkage disequilibrium
LEPR: leptin receptor
MAF: minor allele frequency
NAMPT: nicotinamide phosphoribosyltransferase
OR: odds ratio
SD: standard deviation
SE: standard error
SNP: single nucleotide polymorphism
TDCOB: The Danish Childhood Obesity Biobank
